# Effects of noise on health-related quality of life: The roles of outdoor noise, indoor noise, and noise sensitivity

**DOI:** 10.1038/s41370-025-00816-9

**Published:** 2025-10-28

**Authors:** Sang Hee Park, Pyoung Jik Lee

**Affiliations:** 1 Korea Institute of Civil Engineering and Building Technology, Goyang, South Korea; 2 Acoustics Research Unit, School of Architecture, University of Liverpool, Liverpool, UK

**Keywords:** Noise, Transportation, HRQoL, Quality of Life, Sensitivity, Road traffic

## Abstract

**Background:**

The relationship between transportation noise and health-related quality of life (HRQoL) has been established, but its specific effects on residents of multi-story buildings, who are also exposed to neighbour noise, require further investigation.

**Objective:**

This study sought to investigate the effects of transportation noise on HRQoL among adults residing in such buildings.

**Methods:**

A cross-sectional study was conducted with 400 participants recruited from four apartment complexes. Transportation noise was measured over a 24-h period, and the resulting data were used to create noise maps. HRQoL was assessed using the RAND-36, which measures physical (PCS) and mental (MCS) component summaries.

**Results:**

The results revealed significant negative associations between outdoor noise exposure (overall, road traffic, and railway) and both PCS and MCS, with road traffic noise showing the strongest effects. Noise sensitivity, indoor acoustic satisfaction, and outdoor noise annoyance were identified as significant effect modifiers, with higher sensitivity and annoyance levels exacerbating the negative impacts of noise on HRQoL.

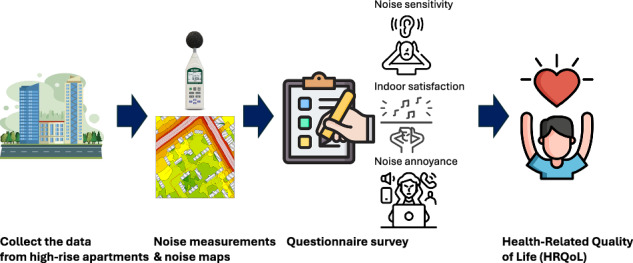

**Impact statement:**

- This study contributes to the growing body of evidence on the health effects of noise pollution by addressing several underexplored aspects. First, it examines the differential impacts of road traffic and railway noise on HRQoL, providing insights into the relative contributions of these noise sources. Second, it investigates the role of noise sensitivity and indoor acoustic satisfaction as potential modifiers of noise-related health outcomes, offering valuable information for targeted interventions. Finally, the study’s focus on urban apartment residents highlights the unique challenges faced by this population, informing policies and building design strategies aimed at improving residential environments and public health.

## Introduction

Noise pollution is a pervasive environmental stressor increasingly recognised for its adverse effects on human health and well-being. Urban residents, particularly those in densely populated areas, are frequently exposed to noise from transportation sources such as road traffic and railways. Chronic exposure to such noise has been linked to a range of health outcomes, including sleep disturbance [1], cardiovascular diseases [2], and impaired mental health [3]. The impact of noise on health-related quality of life (HRQoL) has received significant attention, as HRQoL includes both the physical and mental dimensions of well-being, reflecting an individual’s overall health status.

More broadly, Quality of Life (QoL) is a multidimensional construct that covers various domains reflecting individuals’ overall well-being and life satisfaction. Several international frameworks systematically capture QoL, including the World Health Organization Quality of Life (WHOQOL) [4], the Organisation for Economic Co-operation and Development (OECD) Better Life Index [5], the European Union Statistics on Income and Living Conditions (EU-SILC) [6], and the United Nations Development Programme (UNDP) Human Development Index (HDI) [7]. These frameworks include indicators such as income, education, employment, social relationships, environmental conditions, and civic engagement. While their emphases may vary, health consistently emerges as a central domain. WHOQOL explicitly includes physical and psychological health, while the OECD and EU-SILC incorporate both objective (e.g., life expectancy, activity limitations) and subjective (e.g., self-rated health) health measures. The HDI, though narrower in scope, reinforces health’s fundamental role through its focus on life expectancy. Together, these frameworks highlight that health is not merely one domain among many, but a foundational element that enables participation in education, work, social relationships, and civic life.

Within this broader QoL framework, HRQoL focuses specifically on key domains such as physical, psychological, and social functioning, as well as perceptions of general health [8]. HRQoL is influenced by a wide range of environmental factors. For example, studies have shown that access to green spaces and natural environments is positively associated with HRQoL, particularly in urban settings [9, 10]. Green spaces have been linked to improved mental health, reduced stress, and enhanced social interactions, all of which contribute to higher HRQoL scores. Similarly, air quality has been identified as a critical determinant of HRQoL, with exposure to air pollution associated with reduced physical functioning and increased respiratory and cardiovascular morbidity [11, 12]. Boudier, Markevych et al. [13] also reported that individuals exposed to higher air pollution exhibited lower mental components of HRQoL.

Among environmental stressors, noise exposure has been shown to have particularly detrimental effects on HRQoL. Transportation noise, including road traffic and railway noise, is a major source of environmental noise in urban areas and has been consistently associated with reduced HRQoL scores. The mechanisms through which noise affects HRQoL are multifaceted. Noise exposure can lead to sleep disruption, increased stress levels, and reduced restorative capacity, all of which can impair physical and mental health [14]. Additionally, noise-induced annoyance has been identified as a key mediator of the relationship between noise exposure and HRQoL, with higher levels of annoyance associated with poorer HRQoL outcomes [15].

Numerous studies have demonstrated that higher levels of road traffic noise are associated with lower scores on HRQoL measures. For instance, Basner, Babisch et al. [14] found that individuals exposed to high levels of road traffic noise reported significantly lower HRQoL scores, particularly in domains related to mental health and vitality. Similarly, Miedema and Vos [16] demonstrated a dose-response relationship between road traffic noise exposure and self-reported annoyance, which, in turn, was associated with poorer HRQoL outcomes. Railway noise has also been linked to adverse health effects, though the evidence is somewhat mixed. Öhrström, Skånberg et al. [17] found that railway noise was associated with increased sleep disturbance and reduced overall well-being, particularly among individuals living in close proximity to railway lines. However, some studies suggest that the impact of railway noise on HRQoL may be less significant compared to road traffic noise, possibly due to differences in noise characteristics and individual adaptation [17, 18].

Individual differences in noise sensitivity play a critical role in shaping the health impacts of noise exposure. Noise sensitivity, defined as an individual’s predisposition to perceive and react negatively to noise, has been shown to amplify the adverse effects of noise on mental health [19]. For example, individuals with high noise sensitivity report greater annoyance and stress in response to noise, even at lower exposure levels [15]. Lee, Park et al. [20] classified residents of apartment buildings into low and high noise sensitivity groups and compared their blood pressure levels, finding that noise-sensitive residents showed greater blood pressure levels than others. These findings suggest that noise sensitivity may play a significant moderating role in noise-related health outcomes, though its role in shaping HRQoL remains underexplored.

In addition to outdoor noise, indoor noise from neighbours and the construction year of the building have emerged as significant contributors to noise disturbance and health symptoms [21]. In the context of the present study, “indoor noise” refers to sounds originating from within the building but outside the individual dwelling unit, such as noise from adjacent apartments or shared building systems, whereas “outdoor noise” refers to environmental noise sources external to the building envelope, such as road traffic and railway noise. Satisfaction with the indoor acoustic environment has been identified as a key factor in mitigating the negative effects of outdoor noise, yet its role in shaping HRQoL remains poorly understood. For example, Evans, Bullinger and Hygge [22] found that individuals who reported higher satisfaction with their indoor acoustic environment were less likely to experience noise-related health complaints, even in the presence of high outdoor noise levels. Chan, Wu et al. [23] recently reported that acoustic quality in the dwelling is significantly correlated with all four domains of quality of life – physical health, psychological well-being, social relationships, and environmental conditions – as measured by the World Health Organization Quality of Life - Brief Version (WHOQOL-BREF). Furthermore, Jensen, Rasmussen and Ekholm [24] demonstrated that neighbour noise annoyance is strongly associated with diverse mental and physical health symptoms. These findings highlight the importance of considering both outdoor and indoor noise exposures in studies of noise and HRQoL.

Despite growing awareness of the health implications of noise pollution, a critical gap remains in understanding how specific noise sources, individual sensitivity to noise, and subjective perceptions of the acoustic environment interact to influence HRQoL. Furthermore, while previous studies have predominantly focused on outdoor noise exposure, the role of indoor noise and its interplay with outdoor noise in shaping HRQoL remains underexplored, particularly in high-rise residential buildings. Therefore, this study seeks to address these gaps by examining the relationships between outdoor and indoor noise exposure, noise sensitivity, and HRQoL among urban apartment residents. The primary objective is to investigate the associations between transportation noise exposure (road traffic and railway) and HRQoL, as measured by the RAND-36 instrument. Additionally, the study aims to explore the moderating effects of noise sensitivity and satisfaction with the indoor acoustic environment on these associations. By doing so, this research provides a comprehensive understanding of how individual and environmental factors jointly influence the impact of noise on health and well-being.

## Materials and methods

### Participants

Adult residents from four apartment complexes were recruited following ethical approval from the University of Liverpool’s ethics committee (approval number: 2318). A total of 400 participants were enroled, with hundreds recruited from each complex through advertisements placed in the management office buildings. All participants provided informed consent and completed individual questionnaire surveys in private rooms located within the management offices. Participants received a voucher equivalent to 10,000 KRW for their time and effort.

As listed in Table 1, the study involved a well-balanced gender distribution, with 48% male participants. The average age was 42.9 years, and most participants held college or university degrees. The average length of residence was around 85 months (7 years and 1 month), and most participants indicated that their windows did not directly face the street. Approximately half were employed full-time, and the majority reported an annual household income between £26,660 and £39,993 *-* figures that are above the 2023 national median household income in Korea. Additionally, the size of their homes ranged from 52 m² to 126 m². More than half of the participants responded that noise from footsteps, whether from children or adults, was the most common indoor noise source. The surveys and noise measurements were conducted in November, when outdoor temperatures ranged from -1.3°C to 14.8°C. Given these cool conditions, it is unlikely that windows were frequently opened during the study period, thereby minimising the influence of window-opening behaviour on indoor noise exposure.

**Table 1:** Characteristics of the participants.

Characteristics	
Gender (*n*)	
Male	192 (48%)
Female	208 (52%)
Age (mean ± *SD*)	42.9 ± 10.5
Education (*n*)	
High school	73
College / university	293
Postgraduate or above	34
Occupation (*n*)	
Full-time	206
Part-time	58
Self-employed	28
Student	35
Homemaker	69
Unwaged / retired	3
Other	1
Annual household income (1£ ≈ 1,500 KRW)	
Less than £13,327	3
Between £13,327 and £19,993	38
Between £19,993 and £26,660	66
Between £26,660 and £33,327	111
Between £33,327 and £39,993	104
Higher than £39,993	78
Length of residence (in months; mean ± *SD*)	85.4 ± 62.8
Floor area (i.e. residence size) (m^2^)	52-126
Window orientation (*n*)	
Directly facing the street	91
Not directly facing the street	309
Noise sensitivity (mean ± *SD*)	79.4 ± 13.3
Outdoor noise annoyance (mean ± *SD*)	4.5 ± 2.3
Indoor noise	
Dominant source (*n*)	154
Footsteps: children	100
Footsteps: adults	49
Furniture scraping	50
Dropped items	25
Door banging	22
Plumbing system	
Child(ren) living upstairs (*n*)	218
Yes	114
No	68
Don’t know	5.6 ± 2.8
Indoor noise satisfaction (mean ± *SD*)	

**Fig. 1:**
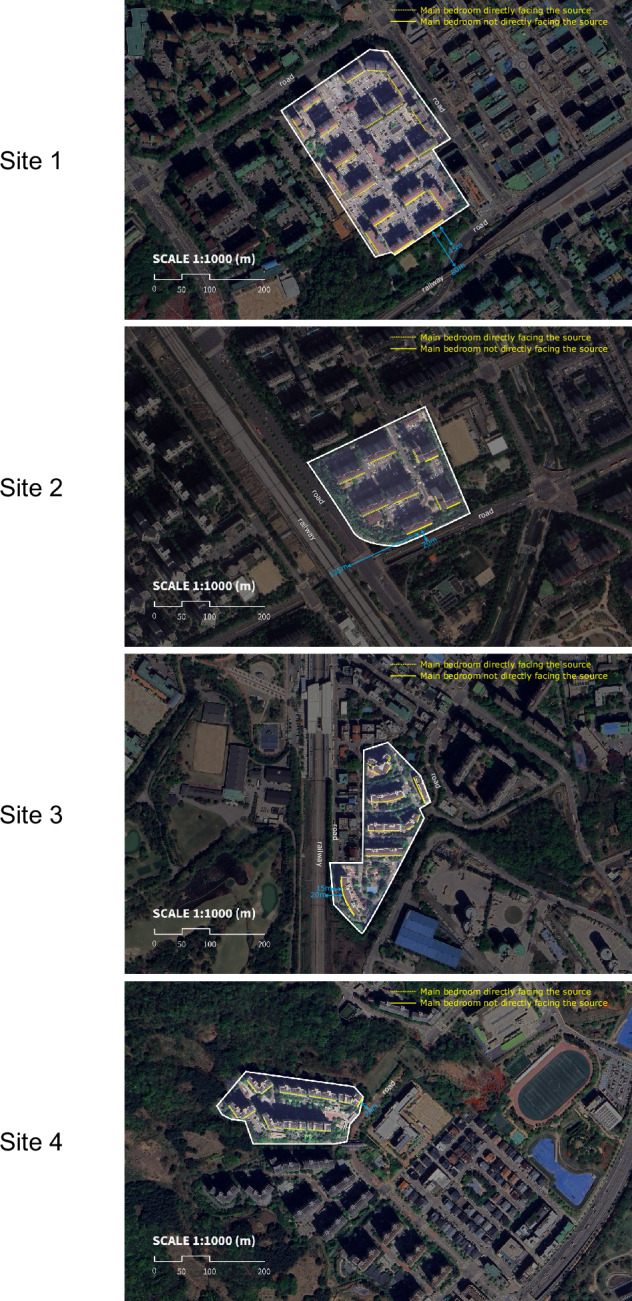
Spatial layout and orientation of study sites relative to major transportation noise sources. Aerial views of Sites 1-4 at 1:1000 scale (m) showing apartment complexes and their proximity to major roads and railways. Dashed lines indicate main bedrooms directly facing noise sources; solid lines indicate main bedrooms not directly facing the sources.

The selection of apartment complexes was based on various factors influencing both indoor and outdoor noise exposure. These factors included proximity to transportation noise sources (roads and railways), building age, and floor thickness. Table 2 provides detailed information about each site. All sites except Site 4 were exposed to noise from both road traffic and railways. Site 4, the newest complex (built in 2014), was only exposed to road traffic noise. In contrast, Site 1, the oldest complex (built in 1994), had the largest number of residences across 21 buildings. The other sites had fewer buildings (7 or 8) and a smaller number of residences, ranging from 262 to 583. Floor thickness, a crucial factor in sound insulation, varied between 150 mm and 210 mm across the different sites. Site 1 was included despite having a larger number of buildings and residences compared to other sites because it represents a typical high-density urban residential environment commonly found in Korea. Including this site allowed the study to capture a broader range of building densities, construction years, and noise exposure profiles, thereby enhancing the representativeness of the sample for urban apartment dwellers. Similarly, Site 4, although exposed only to road traffic noise, was included to provide contrast with the other sites that were affected by both road and railway noise. Its inclusion enabled us to explore HRQoL outcomes under a single-source noise condition and added to the variability of building characteristics within the sample, thereby improving the generalisability of the findings. Figure 1 illustrates the apartment complexes and their surrounding major noise sources (railway and roads), indicating whether the main bedroom windows face these noise sources. For each complex, one sample building’s distances from both the railway and roads are shown.

**Table 2:** Information of the sites.

	Site 1	Site 2	Site 3	Site 4
Transportation noise	Road, railway	Road, railway	Road, railway	Road
Construction year	1994	2002	2009	2014
Number of buildings	21	7	7	8
Number of residences	1827	583	262	522
Highest floor level	25	23	15	18
Slab thickness (mm)	150	150	210	180
Exterior wall thickness (mm)	180	200	200	200
Partition wall thickness (mm)	150	150	150	150
Window type	Aluminium double glazing	Aluminium double glazing	PVC double glazing	PVC double glazing
Fenestration ratio	0.22 ~ 0.30	0.26 ~ 0.29	0.33 ~ 0.38	0.26 ~ 0.33

### Exposure assessment

Twenty-four-hour noise measurements were conducted at the rooftops of 18 out of 43 buildings using Class 1 sound level meters. A-weighted equivalent sound pressure levels were recorded every minute with fast time weighting (*L*_Aeq,1-min_) and used to calculate day-evening-night noise levels (*L*_DEN_). Measured *L*_DEN_ values ranged from 39 to 65 dB across the four sites. Noise maps were generated using SoundPLAN software (version 7.4), incorporating noise measurement data and traffic flow data from the Korean Government. The highest road traffic volume at Site 1 was 40,555 vehicles per day on the busiest roads, whereas Site 4 recorded the lowest volume, with 17,581 vehicles per day. Peak road traffic volumes occurred in the early morning between 7 am and 9 am, and in the evening between 5 pm and 6 pm across all sites. The number of trains per day was 267, 457, and 448 for Sites 1 to 3, respectively. Trains operated from around 6 am to 12 am at all sites, with no metro services running during the nighttime. Hourly train flows did not vary significantly, although peak times were observed from 7 am to 8 am, and from 5 pm to 6 pm. A strong agreement (within 3 dB) was observed between predicted and measured *L*_DEN_. To assess individual noise exposure, *L*_DEN_ levels for each housing unit were predicted by simulating sound propagation to building facades. Additionally, separate noise level predictions (*L*_DEN_, *L*_Day_, and *L*_Night_) were conducted for road traffic and railway noise sources at Sites 1-3, and for road traffic noise at Site 4. These noise level estimates were used to investigate the potential impact of noise exposure on HRQoL.

The noise levels significantly varied across different floors, even within the same building. For instance, the *L*_DEN_ ranged from 33.5 dB to 50 dB across floors in a specific building at Site 1. Sound level measurements were conducted using multiple sound level meters operating simultaneously at each site. Specifically, three meters were deployed at Site 1, while five meters were used at Sites 2, 3, and 4. The noise data collection at each site was completed over two to three days. While most measurements were conducted on weekdays, some took place over weekends to capture potential variations in noise patterns. All measurements were carried out under dry weather conditions to ensure data reliability. The average temperature during the measurement period ranged from 7.5°C to 14.8°C for Sites 1–3, and from -1°C to 3.2°C for Site 4, which was measured later in November.

### Instruments

The HRQoL was assessed using the RAND-36 (https://www.rand.org/pubs/reprints/RP971.html). The RAND-36 measures eight domains of HRQoL: physical functioning, role limitations resulting from physical health problems, bodily pain, social functioning, general mental health, role limitations resulting from emotional problems, vitality, and general health problems. The eight domains were combined to create two separate summary scores: the physical component summary (PCS) and the mental component summary (MCS) scores. The PCS is comprised of four domains: physical functioning, role-physical, bodily pain, and general health. The MCS is comprised of four domains: vitality, social functioning, role-emotional, and mental health.

Noise sensitivity was assessed using the 21-item Weinstein’s noise sensitivity scale [19]. Noise annoyance caused by outdoor transportation noise was assessed using an 11-point numerical scale (0 = *‘not at all’* and 10 = *‘extremely’*), as recommended by ISO/TS 15666 [25]. Participants were asked: “Thinking about the last 12 months, when you are here at home, how much does noise from outside annoy you?” Indoor noise satisfaction was also measured using an 11-point numerical scale with the following instruction: “Thinking about the last 12 months, when you are here at home, how much are you satisfied with indoor noise from neighbours?” Additionally, general socio-demographic information, such as age, gender, annual household income, education level, employment status, floor level, length of residence, and the presence of children living upstairs, was collected from the participants.

### Statistical analysis

Statistical analyses were performed using SPSS for Windows (version 22.0) on the variables listed in Table 1 and the predicted outdoor noise levels. Spearman’s rank correlation coefficients were used to assess correlations between variables, such as annoyance, PCS, and MCS. Independent samples t-tests and Mann-Whitney U-tests were employed to compare differences between groups (e.g., sensitive and less sensitive groups), depending on the data distribution. Multiple linear regression models were constructed to identify significant predictors of the PCS and MCS scores. Variables that exhibited significant associations with PCS and MCS in univariate analyses were included in the regression models. Statistical significance was determined at the 0.05 level (*p* < 0.05). Gardner-Altman plots were used to quantify effect sizes and assess the precision of the statistical analyses [26, 27]. In the figures, the left section reports all individual measurements as a swarmplot to display the underlying distribution. The effect size is reported on the right section, with the mean difference between the groups depicted as a black dot and 95% bootstrap confidence intervals calculated from nonparametric sampling of the collected data, shown by the shaded curve and whiskers.

## Results

Table 3 shows Spearman’s correlation coefficients between PCS and MCS and the tested variables. First, several variables related to personal characteristics showed significant correlations with PCS and MCS. Age was negatively correlated with MCS, while the length of residence and self-reported noise sensitivity were negatively correlated with both PCS and MCS. House ownership, on the other hand, showed positive correlations with both PCS and MCS. Second, variables related to dwelling characteristics were also significantly correlated with PCS and MCS. Building age was negatively correlated with both PCS and MCS, while distance from the traffic road was positively correlated with both. The MCS was negatively correlated with floor area but positively correlated with window orientation. Third, among the indoor noise characteristics, self-reported indoor noise satisfaction was positively correlated with both PCS and MCS. Additionally, PCS showed a positive correlation with slab thickness, whereas MCS was negatively correlated with the presence of children living upstairs. Fourth, all outdoor noise levels were significantly correlated with MCS. Similarly, overall noise levels and road traffic noise levels were correlated with PCS, while the correlations between railway noise levels and PCS were not significant. Last, self-reported noise annoyance ratings showed significant correlations with both PCS and MCS, indicating that participants who perceived outdoor noise as more annoying were more likely to have/experience lower PCS and MCS.

**Table 3:** Spearman’s correlation coefficients of PCS and MCS with the tested variables (**p < 0.01 and *p < 0.05).

	PCS	MCS
Personal characteristics		
Age	−0.073	−0.287**
Gender	−0.020	0.016
Length of residence (in months)	−0.133**	−0.150**
Education	.039	−0.062
Occupation	−0.004	−0.045
Annual household income	−0.073	−0.040
House ownership	0.204**	0.238**
Child(children) living with	0.087	0.055
Self-reported noise sensitivity	−0.135**	−0.217**
Dwelling characteristics		
Building age (in months)	−0.183**	−0.151**
Floor of the house	−0.031	−0.050
Floor area (i.e. residence size) (m^2^)	−0.058	−0.104*
Window orientation	0.102*	0.217**
Distance from the traffic road (m)	0.180**	0.258**
Distance from the railway (m)	0.012	0.113
Indoor noise		
Slab thickness (mm)	0.145**	0.070
Child(ren) upstairs	−0.059	−0.114*
Noise source	.020	−0.085
Time of noise	0.048	0.035
Self-reported indoor noise satisfaction	0.159**	0.208**
Outdoor noise		
Overall_*L*_DEN_	−0.268**	−0.295**
Overall_*L*_Day_	−0.270**	−0.293**
Overall_*L*_Night_	−0.258**	−0.303**
Road traffic noise_*L*_DEN_	−0.264**	−0.283**
Road traffic noise _*L*_Day_	−0.263**	−0.282**
Road traffic noise _*L*_Night_	−0.264**	−0.294**
Railway noise_*L*_DEN_	−0.013	−0.160**
Railway noise_*L*_Day_	−0.012	−0.163**
Railway noise_*L*_Night_	−0.013	−0.156**
Self-reported noise annoyance	−0.274**	−0.364**

Table 4 indicates the associations between noise exposures and PCS and MCS obtained from the regression analysis. A 5-dB increase in all noise sources (overall, road traffic, and railway) and noise exposures (*L*_DEN_, *L*_Day_, and *L*_Night_) were significantly associated with both PCS and MCS, except for railway noise levels and PCS. All standardised regression coefficients (*β*) were negative, indicating that both PCS and MCS decrease with increasing noise levels. Overall and road traffic noise levels had similar associations with PCS and MCS. For example, the *β* of overall noise level for 24 h (*L*_DEN_) was estimated to be −0.33 for PCS and −0.30 for MCS. The 95% confidence interval of β for *L*_DEN_ lies between −0.96 and −0.44 for PCS, and between -1.03 and −0.47 for MCS. Railway noise, however, showed significant associations only with MCS, and the β of railway noise was smaller than those of overall and road traffic noise levels. For instance, the *β* of the 24-h railway noise (*L*_DEN_) for MCS was −0.18 (95% CI: −0.83 to −0.19), whereas the *β* of overall noise for 24 h (*L*_DEN_) for MCS was −0.30 (95% CI: -1.03 to −0.47). Furthermore, the associations between noise exposures (*L*_DEN_, *L*_Day_, and *L*_Night_) and PCS and MCS were found to be comparable. Therefore, to simplify the analysis, only *L*_DEN_ was used for subsequent investigations into the relationship between noise exposure and PCS and MCS.

**Table 4:** Association between PCS and MCS and noise levels; estimated changes in PCS and MCS for a 5-dBA increment in noise level.

	PCS^a^						MCS^b^					
	β/5 dBA		95% CI		*p*-value		β/5 dBA		95% CI		*p*-value	
Overall noise												
*L*_DEN_	−0.33	−0.96		−0.44		0.000	−0.30	−1.03		−0.47		0.000
*L*_Day_	−0.32	−0.93		−0.42		0.000	−0.29	−0.99		−0.45		0.000
*L*_Night_	−0.33	−1.07		−0.48		0.000	−0.30	−1.15		−0.52		0.000
Road traffic noise												
*L*_DEN_	−0.29	−0.84		−0.38		0.000	−0.25	−0.88		−0.37		0.000
*L*_Day_	−0.29	−0.95		−0.43		0.000	−0.26	−1.02		−0.44		0.000
*L*_Night_	−0.30	−0.97		−0.45		0.000	−0.25	−1.00		−0.42		0.000
Railway noise												
*L*_DEN_	−0.05	−0.44		0.19		0.44	−0.18	−0.83		−0.19		0.002
*L*_Day_	−0.04	−0.42		0.22		0.53	−0.18	−0.82		−0.18		0.003
*L*_Night_	−0.06	−0.47		0.17		0.37	−0.18	−0.82		−0.18		0.002

^a^The models for testing PCS were adjusted for length of residence, house ownership, size of the house, child(ren), and child(ren) living upstairs.

^b^The models for testing MCS were adjusted for length of residence, house ownership, age, and child(ren) living upstairs.

Three variables were considered as effect modifiers affecting the associations between noise exposure and PCS and MCS: (1) noise sensitivity, (2) indoor acoustic environment satisfaction, and (3) total outdoor noise annoyance. To investigate the impacts of modifiers, participants were divided into two groups based on their noise sensitivity scores and annoyance ratings. The mean noise sensitivity score (1 ~ 126) was 79.3 (SD = 13.3). Participants whose noise sensitivity scores were ≤ 79.3 were classified as the low noise sensitivity group (*N* = 204), while those with noise sensitivity scores > 79.3 were classified as the high noise sensitivity group (*N* = 196). Similarly, the mean indoor acoustic environment satisfaction (0 ~ 10) was 5.6 (SD = 2.8). Participants with ratings ≤ 5.6 were categorised as the low satisfaction group (*N* = 153), while those with ratings > 5.6 were categorised as the high satisfaction group (*N* = 247). Furthermore, the mean total outdoor noise annoyance rating (range: 0-10) was 4.5 (SD = 2.3). Participants with ratings ≤ 4.5 were categorised as the low noise annoyance group (*N* = 207), while those with ratings > 4.5 were categorised as the high noise annoyance group (*N* = 193).

**Fig. 2:**
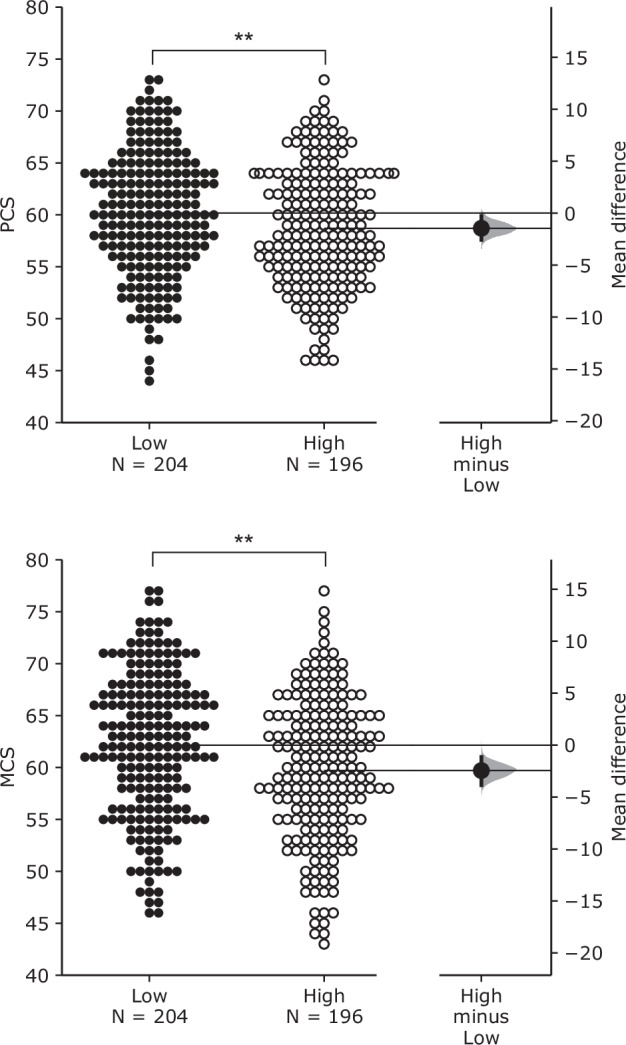
Effect of noise sensitivity on PCS (top) and MCS (bottom). Gardner–Altman estimation plots show the effect size as the mean difference between low and high noise sensitivity groups. Both groups (low and high) are plotted against the left axis. The effect size (black dot) is plotted against the right axis with a 95% confidence interval (indicated by the ends of the vertical error bar) and bootstrap resampling distribution (displayed as a grey curve). **p < 0.01.

PCS and MCS results for low and high noise sensitivity groups are plotted in the Gardner-Altman plots of Fig. 2. For both PCS and MCS, the high noise sensitivity group showed lower ratings than the less sensitive group. For PCS, the mean difference between the groups was -1.46 (95% CI: -2.58 to −0.281), while for MCS, the mean difference between the groups was -2.45 (95% CI: -3.85 to -1.14). Independent t-tests confirmed that the PCS and MCS of the high noise sensitivity group were significantly lower than those of the low noise sensitivity group (*p* < 0.01 for both). The modifying effects of noise sensitivity on the associations between noise exposure and PCS and MCS are listed in Table 5. Overall and road traffic noise showed significant associations between a 5-dB increase and PCS and MCS, while railway noise did not show significant results for either PCS or MCS. High noise sensitivity groups had slightly greater regression coefficients than low noise sensitivity groups for both overall and road traffic noise, but the differences between them were minimal. In general, overall noise showed slightly stronger associations between a 5-dB increase and PCS and MCS than road traffic noise.

**Table 5:** Modifying effects of noise sensitivity (low and high) on the associations between PCS, MCS, and noise levels; estimated change in PCS and MCS for a 5-dBA increment in noise level for 24 h.

*L*_DEN_	PCS^a^				MCS^b^			
	β/5 dBA	95% CI		*p*-value	β/5 dBA	95% CI		*p*-value
Overall noise								
Low noise sensitivity	−0.31	−1.08	−0.30	0.001	−0.27	−1.14	−0.33	0.000
High noise sensitivity	−0.33	−1.01	−0.30	0.000	−0.31	−1.09	−0.32	0.000
Road traffic noise								
Low noise sensitivity	−0.27	−0.95	−0.26	0.001	−0.25	−1.02	−0.28	0.001
High noise sensitivity	−0.30	−0.89	−0.26	0.000	−0.26	−0.95	−0.26	0.001
Railway noise								
Low noise sensitivity	0.02	0.02	0.23	0.82	−0.12	−0.80	0.13	0.15
High noise sensitivity	−0.09	−0.66	0.25	0.37	−0.13	−0.85	0.11	0.13

^a^The models for testing PCS were adjusted for length of residence, house ownership, size of the house, child(ren), and child(ren) living upstairs.

^b^The models for testing MCS were adjusted for length of residence, house ownership, age, and child(ren) living upstairs.

**Fig. 3:**
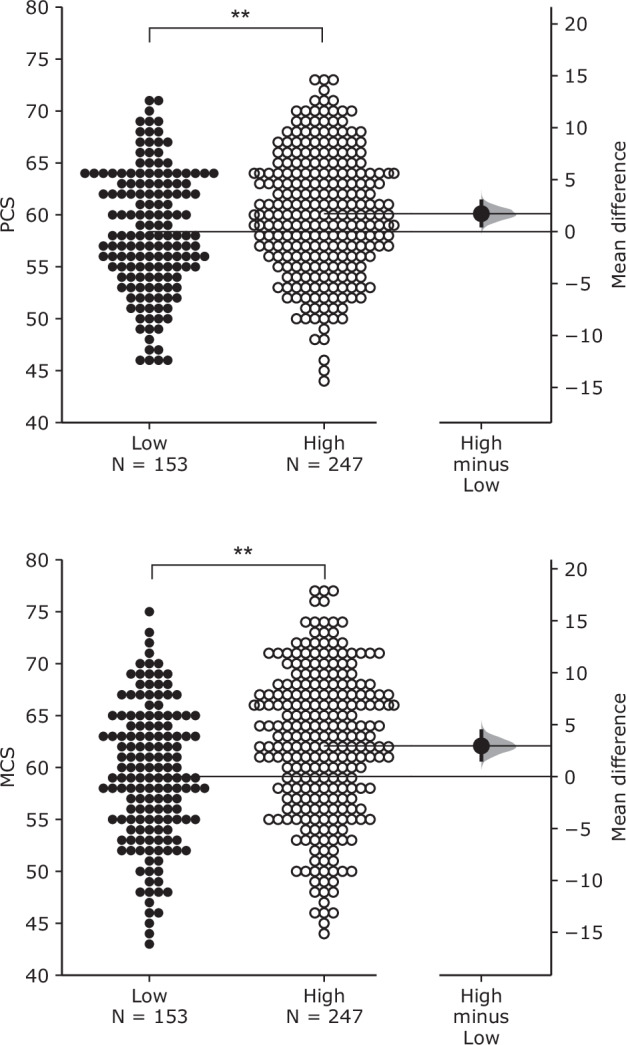
Effect of indoor noise satisfaction on PCS (top) and MCS (bottom). Gardner–Altman estimation plots show the effect size as the mean difference between low and high satisfaction groups. Both groups (low and high) are plotted against the left axis. The effect size (black dot) is plotted against the right axis with a 95% confidence interval (indicated by the ends of the vertical error bar) and bootstrap resampling distribution (displayed as a grey curve). **p < 0.01.

Figure 3 shows the Gardner-Altman plots for PCS and MCS across indoor acoustic environment satisfaction groups. Greater PCS and MCS were observed from the high satisfaction group. The mean differences between low and high satisfaction groups were 1.73 (95% CI: 0.588 to 2.92) and 2.95 (95% CI: 1.63 to 4.37) for PCS and MCS, respectively. The differences between the groups were statistically significant (*p* < 0.01 for both). Table 6 shows the associations between noise exposure and PCS and MCS for low and high satisfaction groups. Similar to the noise sensitivity results, overall and road traffic noise showed significant associations between a 5-dB increase and PCS and MCS. With regard to railway noise, only the low satisfaction group had a significant association with MCS. Overall noise showed slightly greater regression coefficients than road traffic noise, while PCS had slightly stronger coefficients than MCS for both overall and road traffic noise.

**Table 6:** Modifying effects of indoor acoustic environment satisfaction (low and high) on the associations between PCS, MCS, and noise levels; estimated change in PCS and MCS for a 5-dBA increment in noise level for 24 h.

*L*_DEN_	PCS^a^				MCS^b^			
	β/5 dBA	95% CI		*p*-value	β/5 dBA	95% CI		*p*-value
Overall noise								
Low satisfaction	−0.30	−1.07	−0.26	0.002	−0.27	−1.08	−0.24	0.002
High satisfaction	−0.38	−1.11	−0.43	0.000	−0.34	−1.21	−0.48	0.000
Road traffic noise								
Low satisfaction	−0.26	−0.92	−0.18	0.004	−0.20	−0.86	−0.10	0.014
High satisfaction	−0.35	−1.00	−0.41	0.000	−0.31	−1.11	−0.45	0.000
Railway noise								
Low satisfaction	−0.14	−0.14	−1.36	0.18	−0.26	−1.19	−0.22	0.005
High satisfaction	0.05	−0.31	0.55	0.58	−0.12	−0.76	0.10	0.13

^a^The models for testing PCS were adjusted for length of residence, house ownership, size of the house, child(ren), and child(ren) living upstairs.

^b^The models for testing MCS were adjusted for length of residence, house ownership, age, and child(ren) living upstairs.

**Fig. 4:**
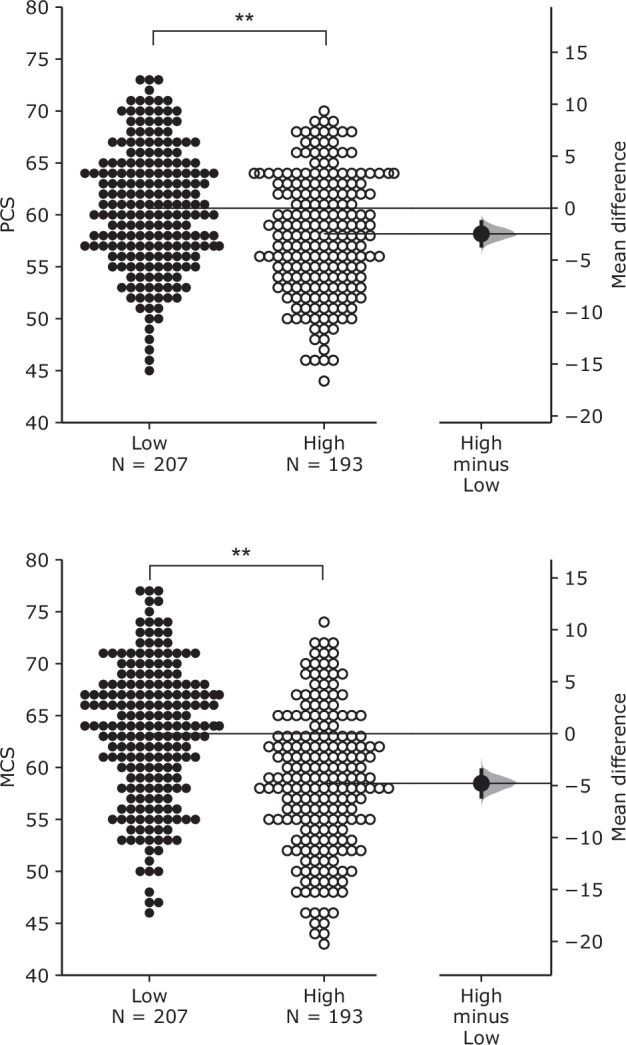
Effect of total outdoor noise annoyance on PCS (top) and MCS (bottom). Gardner–Altman estimation plots show the effect size as the mean difference between low and high annoyance groups. Both groups (low and high) are plotted against the left axis. The effect size (black dot) is plotted against the right axis with a 95% confidence interval (indicated by the ends of the vertical error bar) and bootstrap resampling distribution (displayed as a grey curve). **p < 0.01.

Figure 4 represents the Gardner-Altman plots for PCS and MCS across total outdoor noise annoyance groups. For both PCS and MCS, participants who showed low annoyance ratings due to outdoor noise had higher PCS and MCS ratings than high annoyance groups. The mean differences between low and high annoyance groups were -2.47 (95.0% CI: -3.61 to -1.34) for PCS and -4.77 (95.0% CI: -6.08 to -3.51) for MCS. The associations between noise exposure and PCS and MCS for low and high annoyance groups are listed in Table 7. Compared to other modifiers, the number of significant associations was relatively small and most significant associations were found from PCS. Specifically, PCS had significant associations with a 5-dB increase for overall and road traffic noise. However, MCS showed only two significant associations with overall and road traffic noise, only for low annoyance groups.

**Table 7:** Modifying effects of total outdoor noise annoyance (low and high) on the associations between PCS, MCS, and noise levels; estimated change in PCS and MCS for a 5-dBA increment in noise level for 24 h.

*L*_DEN_	PCS^a^				MCS^b^			
	β/5 dBA	95% CI		*p*-value	β/5 dBA	95% CI		*p*-value
Overall noise								
Low annoyance	−0.26	−1.21	−0.22	0.005	−0.19	−1.08	−0.08	0.024
High annoyance	−0.22	−0.98	−0.20	0.003	−0.10	−0.74	0.13	0.175
Road traffic noise								
Low annoyance	−0.23	−1.15	−0.21	0.005	−0.19	−1.14	−0.11	0.017
High annoyance	−0.25	−0.84	−0.20	0.002	−0.09	−0.56	0.13	0.214
Railway noise								
Low annoyance	−0.08	−0.08	−0.70	0.49	−0.07	−0.74	0.35	0.476
High annoyance	0.03	−0.33	0.49	0.71	−0.14	−0.80	0.03	0.07

^a^ The models for testing PCS were adjusted for length of residence, house ownership, size of the house, child(ren), and child(ren) living upstairs.

^b^The models for testing MCS were adjusted for length of residence, house ownership, age, and child(ren) living upstairs.

## Discussion

### General discussion

The results of this study revealed several key insights. First, among the various factors examined, conditions associated with lower PCS and MCS scores were identified. Regarding housing characteristics, older buildings and shorter distances to traffic roads were associated with lower PCS and MCS scores, consistent with previous studies reporting that perceived housing problems negatively impact PCS and MCS [28]. In terms of individual resident characteristics, a longer length of residence, lower satisfaction with indoor noise, higher noise sensitivity, and greater self-reported noise annoyance were linked to lower PCS and MCS scores. Chimed-Ochir, Ikaga et al. [28] also found that individuals without thermal, acoustic, or lighting-related housing issues reported a better quality of life compared to those experiencing such problems. In particular, some findings aligned with prior studies demonstrating a relationship between HRQoL and noise sensitivity [29–32].

Moreover, a 5-dBA increase in almost all noise sources had a statistically significant impact on both PCS and MCS. The standardised regression coefficients (β) were consistently negative, indicating that as noise levels increased, both physical and mental health deteriorated. This finding supports the established understanding that noise exposure negatively affects quality of life, confirming that residents of multi-family housing buildings exposed to neighbour noise also experience similar effects. The building characteristics previously identified as being associated with lower PCS and MCS - namely, older buildings and closer proximity to roads - are likely due to the fact that these conditions inherently contribute to higher noise exposure. Older buildings, in particular, may allow greater noise transmission due to architectural and construction features such as windows with poor sealing and less effective insulated wall assemblies, which are less effective at soundproofing compared to modern standards. However, these specific features were not directly measured in the current study. Future research should examine detailed building attributes, including window types, wall construction, and insulation specifications, and incorporate direct acoustic measurements in buildings of varying ages to more accurately assess how structural characteristics contribute to indoor noise exposure and their subsequent effects on quality of life.

The correlation and regression analyses constantly showed that railway noise did not have any significant effect on PCS but did impact MCS. In contrast, overall noise and road traffic noise had significant effects on both PCS and MCS. These results imply that road traffic noise has a greater impact on both physical and mental health than railway noise. Road traffic noise is a continuous environmental stressor, characterised by unpredictable variations in traffic volume, honking, acceleration, and deceleration. Furthermore, its persistence during night-time hours, particularly intermittent noise events, can disrupt sleep and trigger chronic physiological stress responses [31, 33]. According to the 2017 Environmental Noise Directive (END) of the European Union, road traffic noise was reported to reach levels adverse to health. Compared to railway noise, road traffic noise has been found to have a stronger association with cardiovascular diseases and premature mortality, highlighting its substantial health burden [34]. Another potential reason for the weak association between railway noise and HRQoL in this study is that some participants were located far from the railway tracks, resulting in significantly lower railway noise levels compared to road traffic noise. For instance, approximately 30% of participants at Site 1 were situated more than 200 m away from the railway.

Previous research has suggested that, for a given noise level, railway noise tends to be less annoying than road traffic noise. This phenomenon, often referred to as the ‘railway bonus’, is attributed to the predictability of railway noise, as trains operate on fixed schedules with limited night-time services, making it easier for nearby residents to anticipate and adapt to the noise. However, the present study found that while railway noise had no significant impact on PCS, it was significantly associated with MCS. Although the impact of road traffic noise on MCS (β = 0.25) was greater than that of railway noise (β = −0.18), the mental health effects of railway noise should not be overlooked. Consistent with previous studies, railway noise has been shown to negatively affect mental health by contributing to stress, fatigue, and anxiety. Although it is more predictable than road traffic noise, it remains a significant environmental stressor that can disrupt concentration and cause psychological discomfort among nearby residents [35].

The results of this study demonstrated that individual response characteristics were as crucial as noise levels or source types in determining health impacts. The study revealed that the high noise sensitivity group showed consistently lower PCS and MCS compared to the low noise sensitivity group. Furthermore, noise-sensitive individuals showed heightened weakness to noise level increases. For instance, when road traffic noise increased by 5-dBA, the low sensitivity group showed changes in PCS and MCS with β = −0.27 and −0.25, respectively, while the high sensitivity group demonstrated more pronounced effects with β = −0.30 and −0.26. This correlation between noise sensitivity and HRQoL aligns with previous research findings [29].

Highly sensitive individuals are more likely to experience adverse health effects even in identical noise environments. Noise sensitivity, as a psychological characteristic that determines varied individual responses to the same noise environment, serves as a critical moderating factor in health-related research [36]. This heightened sensitivity often leads to amplified stress responses and may contribute to long-term psychological distress and physical ailments [27, 36–38]. For example, noise-sensitive individuals exhibited significantly lower EEG alpha wave activities compared to less sensitive groups [27], indicating higher cortical arousal and stress levels, while also demonstrating greater systolic and diastolic blood pressure levels than their counterparts [20].

The present study also confirmed that subjective satisfaction with the indoor acoustic environment significantly influenced both PCS and MCS, with higher satisfaction levels correlating positively with quality of life. Indoor acoustic satisfaction includes not only experienced noise levels but also spatial acoustic characteristics and overall residential environment assessment. The positive correlation between indoor acoustic satisfaction and higher PCS and MCS aligns with previous studies emphasising the impact of subjective environmental evaluation on health outcomes [39]. The relationship between satisfaction and the health effects of noise appears to differ from that of noise sensitivity and noise annoyance. While lower noise sensitivity and lower noise annoyance had a weaker regression coefficient to noise increases, suggesting that individuals less affected by noise tend to experience less deterioration in their quality of life, the opposite trend was observed for satisfaction. In particular, individuals with higher satisfaction exhibited a stronger negative impact of noise on PCS and MCS compared to those with lower satisfaction.

There are several potential explanations for this pattern. First, individuals with high satisfaction may have greater expectations regarding their living environment. When unexpected noise disrupts their perceived comfort, the discrepancy between expectation and reality could amplify their negative reaction, leading to a stronger decline in their PCS and MCS [40, 41]. Second, individuals with low satisfaction may have already adapted to suboptimal living conditions, making them less reactive to additional noise disturbances. Their overall lower expectations might buffer them against further reductions in PCS and MCS [40, 42]. Finally, it is also possible that those with low satisfaction already experience lower PCS and MCS, leaving less room for further decline due to noise exposure. These findings suggest that improving indoor environmental quality, beyond mere noise reduction, plays a crucial role in residents’ health [39]. Moreover, considering psychological factors such as expectations and adaptation to environmental stressors is essential in understanding the complex relationship between satisfaction, noise, and well-being.

When examining the regression coefficients for the 5-dBA noise increases across groups with different annoyance levels, the results presented somewhat inconsistent patterns. For overall noise, the low-annoyance group showed PCS and MCS changes of β = −0.26 and −0.19, respectively, while the high-annoyance group demonstrated significant changes only in PCS (β = −0.22). This suggests that overall noise increases had a more substantial impact on the low-annoyance group. However, this trend reversed for road traffic noise, where the low-annoyance group showed PCS and MCS changes of β = −0.23 and −0.25, while the high-annoyance group showed significant changes only in PCS (β = −0.19). Several interpretations of these findings merit discussion. First, overall noise, containing multiple sources beyond road traffic, may have more complex effects on low-annoyance groups. Combined noise sources are likely to amplify health impacts compared to individual sources [14]. In addition, highly annoyed individuals may have already developed noise coping strategies (e.g., closing windows, using soundproof curtains), while less annoyed groups might lack such protective behaviours. These results imply that lower self-reported annoyance does not guarantee protection from adverse health impacts, as cumulative noise exposure can negatively affect health indicators regardless of perceived annoyance levels. Moreover, noise annoyance extends beyond sheer discomfort, relating to long-term stress responses and incorporating factors such as individual noise sensitivity, environmental context, and psychological variables [43, 44]. Therefore, intervention strategies should combine noise reduction measures with approaches addressing residents’ noise perception, such as noise information provision or psychological coping strategies education [45, 46].

### Limitations and future research

This study provides valuable insights into the impact of noise exposure on residents’ health and well-being; however, some limitations should be acknowledged. First, the sample size of participants exposed to railway noise was relatively small. Even among respondents residing in apartment complexes near railway tracks, many were located at a considerable distance from the railway or had their exposure reduced due to intervening buildings. As a result, the results regarding the impact of railway noise should be interpreted with caution. Future studies should recruit a more extensive and diverse sample of railway noise-exposed individuals, particularly those living in closer proximity to railway tracks, to provide a more comprehensive understanding of its effects.

Second, railway noise in South Korea consists of various sources, including metro trains, conventional trains, high-speed trains, and freight trains. Among these, metro is one of the dominant forms of public transportation, and its frequent operations may moderate noise perception of other types of railway noise. Furthermore, railway noise may not only be perceived in terms of acoustic disturbance but also in relation to broader socio-economic factors, such as its impact on property values [47]. It has been suggested that negative attitudes towards railway noise are often linked to concerns about declining housing prices and the perceived inconvenience of living near railway infrastructure [48]. Thus, future studies may further investigate the role of socio-economic factors and personal attitudes in shaping railway noise perception among South Korean residents.

Third, the participants were residents from four high-rise apartment complexes in urban areas, reflecting the predominant housing type in many Korean metropolitan regions. While this sampling frame may not fully represent the broader regional population, particularly individuals residing in low-rise or detached housing, the focus on high-rise apartment dwellers aligns with the study’s aim to examine noise exposure and HRQoL in dense urban residential environments. Accordingly, the participants’ responses and the findings in this study are likely to be relevant to similar urban settings characterised by multi-story housing and high population density. Moreover, this study’s primary focus on urban apartment dwellers may limit the generalisability of the findings to other housing types. Noise insulation characteristics vary across housing structures. The absence of nearby expressways at most study sites may have influenced comparative assessments between railway and road traffic noise. An expanded scope of research is needed to include a broader range of residential settings, including detached housing, to assess how different housing structures influence noise perception and health outcomes.

Fourth, although noise exposure levels were predicted at each façade of dwellings, the actual individual noise levels could differ according to the sound insulation performance of building elements and neighbours’ activities. Several studies have measured noise levels at home using mobile phones and wearable devices [23, 49]; however, the accuracy of these measurements cannot be guaranteed without proper sound calibration [50]. There has been substantial progress in developing smart sensors, so in the future, actual individual noise exposure levels could be used to investigate the impacts of noise on health. In addition, noise levels were predicted using noise maps validated against measurements taken only on the rooftops. This limitation may introduce some uncertainty into the predicted noise levels and their relationship with HRQOL outcomes. Future research should include noise measurements on multiple floors for a more robust validation of the noise predictions and to improve the accuracy of dose-exposure relationships.

Additionally, this study highlights the need to incorporate non-acoustic factors in noise impact assessments. While physical noise exposure levels were measured, individual noise sensitivity, attitudes towards noise sources, and coping strategies were not extensively examined. Given the strong associations between these psychological factors and health outcomes [43, 44, 51], future studies might benefit from employing a multidimensional approach that accounts for both objective measurements of noise exposure and subjective experiences of noise perception.

## Conclusions

This study examined the associations between exposure to outdoor transportation noise and health-related quality of life (HRQoL) among residents of four apartment complexes in Korea. The findings revealed significant negative correlations between noise exposure levels and HRQoL scores. Specifically, increases in transportation noise levels were associated with reductions in both the physical and mental component summary (PCS and MCS) scores of the RAND-36 questionnaire. Overall and road traffic noise levels showed significant associations with both PCS and MCS, whereas railway noise had a smaller impact, primarily affecting MCS. Additionally, the study identified several moderating factors influencing the relationship between noise exposure and HRQoL, including noise sensitivity, satisfaction with the indoor acoustic environment, and total outdoor noise annoyance. Individuals with higher noise sensitivity, lower satisfaction with indoor noise conditions, and greater annoyance from outdoor noise reported lower HRQoL scores. These findings underscore the need for practical noise mitigation strategies to improve the quality of life for urban residents.

## Data Availability

The datasets generated during the current study are available from the corresponding author upon reasonable request.
